# Is it safe to conduct interventional research? In clinical settings working under pressure we must first consider system tolerated avoidable risks in care

**DOI:** 10.3389/frhs.2026.1950035

**Published:** 2026-10-14

**Authors:** Mike English, Charles Vincent

**Affiliations:** 1 Health Systems Collaborative, Nuffield Department of Medicine, University of Oxford, Oxford, United Kingdom; 2 Health Services Unit, KEMRI-Wellcome Research Programme, Nairobi, Kenya; 3 Department of Experimental Psychology, University of Oxford, Oxford, United Kingdom

**Keywords:** intervention, LMIC (low and middle income countries), risk, safety, system thinking, workarounds, working conditions

## Abstract

All health systems, even those offering ‘best practice’ care, routinely expose patients and staff to some possibility of avoidable harm. Building on earlier theory we call these “System Tolerated Avoidable Risks in Care” (STARC), explain how they become normalised and normalised and consider the implications for safety and quality interventions. Clinical settings under pressure become progressively degraded through the accumulation of system-induced workarounds that increase the potential for harm but become accepted as everyday practice. These tolerated, largely unremarked operating conditions and associated risks help explain why quality-improvement efforts, implementation and technology or therapeutic intervention programmes often fail or may even cause further harm. We recommend first assessing how degraded a care system is and directly tackling underlying system deficits to stabilise highly dysfunctional systems before intervention in the worst cases. In other cases, we recommend deploying foundational co-interventions in addition to any specific new intervention when operating conditions manifest clear threats to patient safety. Such planning when considering an intervention we believe will increase the likelihood of success, promote patient safety as an important element of interventions and subsequently provide a more complete understanding of what is needed to support adoption at scale and sustained patient benefit.

## Introduction

Continuously providing perfect health care for all, everywhere is impossible and so exposing patients and staff to some degree of risk is a feature of all health systems (1). Systems differ in their efforts to identify and manage certain risks while implicitly tolerating other risks (2). In this Viewpoint we argue that the actual level of ’System Tolerated Risk’ is a largely unremarked product of all health systems. Highly resourced and effectively managed systems should be associated with lower risks and greater resilience but may still tolerate some unacceptable risks to patients (3). Poorly resourced systems implicitly tolerate exposing patients and staff to higher levels of avoidable risk. In both high and low resource settings risks are exacerbated by poor governance and management, system features can themselves be a consequence of under-resourcing.

We suggest it is critical to understand the nature and extent of System Tolerated Avoidable Risks in Care when planning interventional research. In settings where system conditions include limited resources and staff are under intense pressure the care provided is likely to be variable in quality and even routinely expose patients to a high baseline risk of harm (4). Well-intentioned but context naïve interventions, including the introduction of technologies, new forms of therapy or other improvement interventions, may founder in these circumstances or even cause additional harm by further destabilising the system of care (5). In such settings we go on to argue that foundational (co)interventions to address poor operating conditions and stabilise local systems of care may be needed prior to intervention in some cases or to support interventions to enable their success and subsequent adoption in others.

### The forbidden space and hospital patient safety

Work presented 20 years ago by Amalberti, and colleagues articulated how to some degree all clinical settings operate imperfectly (6). Amalberti and colleagues explain that the inevitable minor deviations from idealised ‘written’ procedures occur in all safety-critical systems (6, 7). These minor deviations are often seen as enabling efficient work (so as a benefit) rather than incurring risks, including by team leaders and managers, even in well-resourced settings offering generally high quality care (2). Most if not all health care settings experience increased pressure, temporarily or permanently, when circumstances change. When health workers must complete tasks in limited time or with inadequate resources while still expected to achieve challenging productivity goals minor deviations may become more pronounced. Under pressure minor deviations from defined ‘best practices’ described in policies and guidelines can gradually becoming more frequent and attain the status of accepted ‘workarounds’ (8). Initially staff may be cognisant that when under greater pressure they cannot adhere strictly to recommended standards of care and that this may elevate risks to patients. Relieving the pressure may reduce reliance on workarounds and result in a return to good practice.

Under sustained pressure, especially if severe, workarounds can become more entrenched and more substantive deviations may emerge that are now more likely to undermine overall quality and safety (6, 9). However, If no obvious harm to patients is immediately apparent, or if systems are unable to monitor harms these deviations or violations of expected standards can come to seem acceptable. In a sense the risks have become invisible.

Amalberti et al. refer to the initial, slightly degraded state as Borderline Tolerated Conditions of Use. Under these conditions the majority of deviations that arise as a result of poor operating conditions go unremarked, but on occasions an accumulation or co-incidence of minor deviations, care deficits and resultant small errors can lead to potentially serious adverse effects or harms (10). At times of high pressure, the system can enter the more dangerous state referred to by Amalberti and colleagues as the ‘Forbidden Space’. In the Forbidden Space the normal limits of acceptable good practice applicable in well-resourced settings are clearly violated (6). This poses immediate and direct risks to patients. The surge in cases of severe illness resulting from the SARS-COV2 pandemic is one global example of an extrinsic system shock resulting in forms of care provided that would normally be considered sub-standard. Even if acute pressures are less pronounced a system lacking sufficient reserves of health worker time or materials (organisational slack) may transition to a new more dangerous operating state as it has minimal resilience (11). Chronic system stresses as well as acute shocks can also result in such transitions. Examples might include long-term system failings that require staff to care routinely for severely ill patients on trolleys in hospital corridors. In a LMIC setting chronic pressures may be exacerbated by acute on pressures. For example, failings in the blood transfusion and clinical governance systems can put junior clinicians in the position of having to choose whether or not to transfuse blood not screened for viral pathogens in a child with life-threatening malarial anaemia after a surge in cases has exhausted steadily declining screened blood stocks (12).

The useful framework developed by Amalberti et al. drew largely on their experience with high-income, well-resourced industries and health care settings (6). In these settings there was little expectation that the Forbidden Space, where the normal limits of acceptable practice are routinely violated, would or should be tolerated as a ‘default setting.’ However, in health systems where infrastructure is poor and there are not enough personnel or it is inevitable that staff will not always be able to offer care that meets globally recommended standards, even if these are developed for low-resource settings (13). Prolonged exposure to working in such a ‘Forbidden Space’ where there is a mismatch between the expected standard and system’s ability to support such a standard may then produce what is sometimes interpreted as the risk-tolerating and risk-taking behaviour of individuals, teams and managers, for example habitual failure to use the WHO Safe Surgery checklist (14). These system produced, risk-taking behaviours can further degrade the system, creating a potentially dangerous escalatory feedback loop in which the risk of harm to patients becomes progressively higher.

We set out arguments below to suggest that conditions typical of the Forbidden Space exist in many LMIC hospital settings as a result of severe often unrelenting system pressure. While not specifically endorsed these conditions and their consequences are nevertheless tolerated policy makers and managers and so normalised by individuals (15).

## Managing ‘everyday crisis’ in low resource settings

Many factors conspire to create severe, unrelenting pressure on those charged with providing clinical care in LMIC settings. Resource gaps often result in overcrowded dilapidated spaces, unstandardised, missing and broken equipment, re-use of ’single-use’ items, medicine shortages including lack of age-specific formulations and much more (16). Resources for patients, including for their personal hygiene, are similarly poor. In the same settings there are often pronounced and important shortages of health workers of all professions. Doctors or clinical officers (clinical assistants/medical assistants) who are still interns or just licensed to practice may in many hospitals have primary responsibility for large numbers of severely ill patients, especially at night, including performance of major surgery (17). Nurses work with extreme patient to staff ratios, with substantial numbers of patients often critically ill (18). These nurses may be charged with providing essential care while using and maintaining equipment they are unfamiliar with and navigating competing management and administrative responsibilities including rationing allocation of existing resources (15). Allied health professionals are very few in number severely restricting access to their expertise, and support staff may not be sufficient to complete even essential tasks such as basic ward cleaning to support infection control (19). These conditions are exacerbated by poorly functioning information systems, poor care coordination and poorly organised patient flow amongst other factors (20). Important consequences of these working conditions include poor inter and intra-professional communication and coordination, abandonment of important professional tasks to students without their careful prior training or supervision and falsification of clinical documentation (18).

Coping with such poor working conditions force staff to constantly ‘make do’ in the face of chronic or acute on chronic demand and resource stresses. Observed from a distance this can be seen as a system expressing some form of everyday resilience (21). But observed more closely this operating state is better described and seen as an ‘everyday crisis’ (Figure 1). Importantly, health professionals in these settings are trained in global and contextually appropriate best practice recommendations. However, the working conditions they encounter often mean they cannot meet their own expectations of providing good care. In fact when working in systems under extreme pressure they learn to normalise the workarounds that have emerged so that some form of care is provided even though they are often aware that there are risks to patients that would be avoidable under different circumstances (15). These working conditions and associated risks are rarely formally acknowledged at system level and the dissonance between learned or expected standards of care and the reality of care and its effects on patients have important effects on health workers themselves who are left to try and make sense, either alone or with peers, of their experience providing sub-standard care that conflicts with their personal and professional values (2, 22). The resulting resigned acceptance of practices that health workers themselves know pose risks to patients is also a form of normalisation and has pervasive effects, contributing to burnout, moral distress and emotional disengagement (15, 23). These consequences for the workforce in turn make it more difficult to achieve any required changes in individual or collective behaviours to support improvement efforts or an intervention.

**Figure 1:**
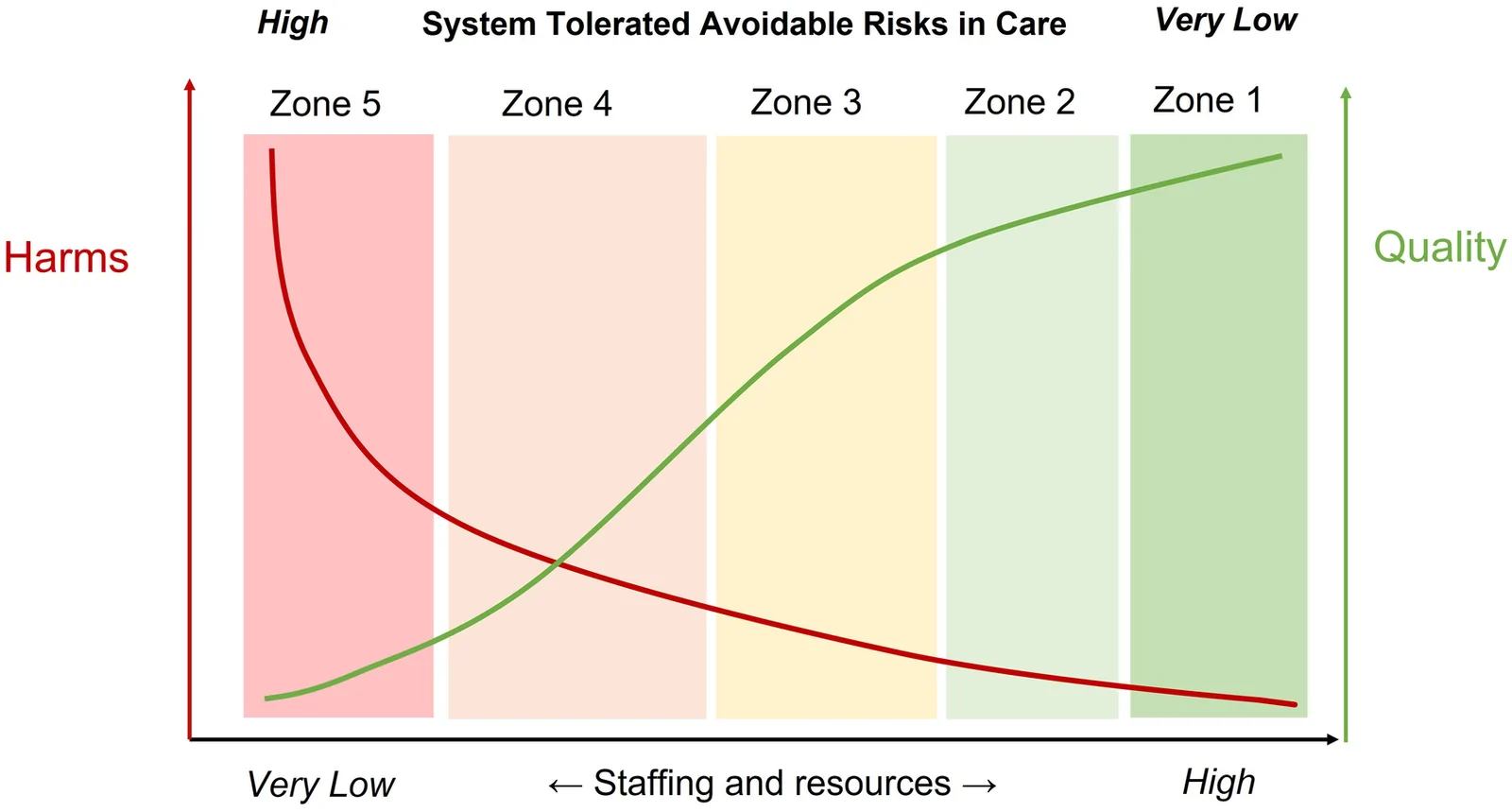
An illustration of how in a high acuity, high workload setting varying workforce capacity and capability (*X* axis) associated with wider resource deficits contribute to variation in quality, risk and consequently potential for harms. Zone 1 or ‘Best Practice’ and Zone 5 (Table 1) illustrate very low and very high potential levels of harm respectively (red line and red/left-hand *Y* axis) and very good/poor overall quality of care (green line and green/right-hand *Y* axis). If operating pressures remain constant foundational (co)interventions will probably be needed to stabilise systems in Zones 4 and 5 before introduction of new interventions while systems in Zone 2 and 3 may still need specific co-interventions. Continued improvements in workforce capacity and capability and resources allied with good leadership and clinical governance, and despite continued high operating pressure, may enable systems to ‘right shift’ and deliver best practice care.

## System tolerated avoidable risks in care

In highly pressured settings with few resources, it is therefore unfair, indeed pointless, to criticise the staff for cutting corners or taking risks. Even when only providing policy-mandated essential services the health care system and the local operating conditions frequently put local managers and health workers in the unenviable position of choosing which courses of action they believe are least likely to harm patients (24). The key driving factor here is the imbalance between the needs for care and the available workforce capacity and capability and material resources. This is typically compounded by a failure of governance. If a system cannot support even a locally defined acceptable standard of care a system response that acknowledges and manages risks is required. In the absence of an appropriate system response the emergence of a structural and normative environment in which violations of acceptable practice and risks of patient harm are common (6, 15, 25). In fact, sustaining some level of service provision in a dysfunctional system often relies, paradoxically, on health workers taking risks that those in authority or peers in much less pressured settings would deem unacceptable. Hence, the notion that this is a Forbidden Space seems misplaced as healthcare workers often have little choice but to manage as best they can in the circumstances imposed on them, and it is neither fair nor useful to focus on (and blame) their inability to provide good care.

We propose instead that we consider these contexts as manifesting to varying degrees System Tolerated Avoidable Risks in Care (STARC). We use this term to emphasise that consciously or not it is the health system that creates and tolerates the level of avoidable risk to which patients and staff are exposed. In Table 1 we suggest how System Tolerated Avoidable Risks in Care exist to varying degrees as a result of human and material resource deficits. We draw on studies from intermediate level neonatal units in low-resource hospital settings to illustrate how care quality and safety can be progressively undermined. Although neonatal units are a specific clinical setting, we suggest this example is pertinent to paediatric and adult care hospital settings based on wider experience. We illustrate the conceptual links between the severity of workforce and wider resource challenges with poor quality care and harm specifically in Figure 1.

**Table 1:** Characterising operating conditions based on the nature and frequency of workarounds or violations of optimal practices. While recognising that the degradation of health care provision is a continuous spectrum, we use examples or workarounds seen on high mortality, intermediate level neonatal care units to illustrate different Zones (and see Figure 1).

	Features of the operating conditions	Examples of workarounds or normalised practices observed in high mortality LMIC neonatal units
Zone 1	Provision of ‘best practice care’ as widely accepted by international professional peers/communities—workarounds represent minor deviations to address the impossibility of providing perfect care even in highly resourced settings. Significant harm rare.	
Zone 2	Workarounds represent planned deviations from explicit standards on the basis of perceived minimal risk. Cumulative errors occasionally sufficient to result in significant harm	STARC +: Patient to staff ratios exceed best practice standards; Re-use of single use equipment after careful local decontamination; temporary reduced frequency of vital signs monitoring for stable patients meeting agreed low-risk criteria in response to acute increase in workloads
Zone 3	Workarounds common, often *ad hoc*, and include some major deviations from optimal care in practices that apparently carry low immediate risk to patients. Cumulative errors contribute to minor harms and uncommon but significant harm to patients.	STARC++: High patient to staff ratios; missed measurements of scheduled vital signs in those with severe illness; missed checks on nasogastric tube positioning prior to feeding; poor hand hygiene; inadequate care documentation; occasional lapses in handover processes between shifts and poor inter-professional communication; limited counselling of mothers
Zone 4	Workarounds include missed steps in higher risk processes/procedures while perceived lower risk processes/procedures may be subject to multiple workarounds or even neglected completely. Potential for significant and more frequent harm to patients resulting directly from specific deviations or from cumulative errors.	STARC+++: Very high patient to staff ratios; Absent/inadequate patient identification checks prior to treatments; inadequate handover processes between shifts and very limited inter-professional communication; missed or delayed diagnostics tests; missed or delayed treatments; absent/inadequate supervision of under-qualified or inexperienced staff; inadequate equipment checks and infection control; lack of counselling of mothers who provide unsupervised forms of care (e.g., nasogastric feeding, giving oral medicines to sick infants)
Zone 5	‘Crisis Care’ when efforts to sustain good practices largely abandoned. Efforts are focused on completion only of highly prioritised tasks considered critical to immediate and urgent patient needs.	Crisis: Extreme patient to staff ratios; Care schedules and structured handover completely disrupted/abandoned (e.g., medical or nursing reviews only performed after patient deterioration identified); babies sharing incubators/cots; invasive procedures performed without appropriate equipment or staff; under-qualified or inexperienced staff work independently without timely supervision; non-compliance with essential infection control practices; mothers have no engagement in monitoring or care with any concerns ignored

Relevance of System Tolerated Avoidable Risks to interventional, improvement and implementation research and programs.

Improvement and implementation research has for many years described barriers in the systems under study that limit improvement or intervention effects. Our contribution, by drawing on ideas from the field of patient safety, is to remind people that system created operating conditions that normalise poor quality care and risks to patients are an important emergent property of the context (1, 6, 8, 15). In particular, we highlight the role of limited workforce capacity and capability in conjunction with wider resource deficits because it seems this feature of LMIC hospital settings is itself so normalised that although aware of it, we continue to fail to take it into account (18). If the normal (tolerated) state of a clinical system is that risks to patient safety are readily apparent and substantial when looked for this has two major implications. First there is an imperative for foundational interventions that improve safety before further intervention. The second is that without foundational co-interventions to address dysfunctional operating conditions local systems are likely to be resistant to change with high risk of intervention failure.

### Understanding and stabilising the existing system

We therefore suggest that a first step in improvement or intervention activities in systems known or expected to be operating under pressure should be an explicit evaluation of the existing quality and safety of care (26). Even if people are familiar with local conditions the question should be asked what degree of System Tolerated Avoidable Risks are apparent (in which Zone does this system reside, Figure 1). Findings may indicate that a pre-intervention period and sometimes new resources are needed to stabilise the existing system to reduce manifest risks of harm and ensure that both staff and system are ready for the proposed intervention. Interestingly, some intervention studies have inadvertently already produced strong indications of the beneficial effect of improving operating conditions. This phenomenon, the ‘infrastructure effect’, refers to the improved outcomes for participants (and even non-participants) in clinical trials as a result of either explicit or inadvertent strengthening of local systems of care (infrastructure). This frequently transient system strengthening often improves the availability of essential resources and provides additional, motivated research staff given time to follow good practice guidelines (27). We argue, as have others, that addressing system deficits through sustained system strengthening should be the first priority, in advance of or together with intervention studies, so that the true effect of an intervention can be assessed as well as for ethical reasons (28).

### Addressing ongoing or emerging system deficits during intervention

The second STARC related implication is that once the system is deemed stable the intervention team should further explore, perhaps with pilot studies, what possible impact their intervention will have on an already fragile system. Will the intervention, for instance, reduce staff time available to provide other aspects of essential care or will a technology be used or maintained by personnel without sufficient competence to ensure safe use? An intervention may improve one aspect of care (the measured focus of the intervention team) while reducing resources and increasing risk to patients elsewhere (potential unintended consequences rarely examined by intervention teams) (29). Thus, even well-intentioned interventions may modify and perpetuate system tolerated avoidable risks in care. In systems under pressure co-interventions that both stabilise systems and promote chances of intervention success should be included as a specific part of studies. These foundational (co)interventions may yield important broad effects for care quality and should then be part of efforts to scale up effective interventions.

## Conclusion

There is a largely unacknowledged, rarely researched but huge burden of morbidity and mortality resulting from unsafe care in LMIC (30). Building on an earlier explanatory framework we show how substantial deviations from good practice are normalised in systems under pressure creating varied states of System Tolerated Avoidable Risks in Care. Greater levels of system degradation are associated with increasing risks of harm. System level workforce and material constraints contribute to these states that are normalised, tolerated and overlooked by key health system actors. The structural conditions that produce System Tolerated Avoidable Risks in Care will also undermine practice change or technological improvement efforts or other interventions limiting expected benefits and increasing the potential of unintended negative consequences. We argue that interventions tackling identified drivers of System Tolerated Avoidable Risks in Care are a foundational requirement to stabilise systems before any intervention when quality and safety are clearly very sub-optimal. In less urgent situations stabilising co-interventions will often be key to support fair evaluation of a more specific intervention and co-interventions should then be part of the package in any subsequent scale-up. Indeed foundational (co)interventions in low-resource settings that improve patient safety may, as in high-income settings, offer a higher return on investment and greater benefit to patients than most other medical interventions (30).

## Data Availability

The original contributions presented in the study are included in the article or in prior published and referenced work, further inquiries can be directed to the corresponding author.
